# Enhancing ICU Outcomes Through Intelligent Monitoring Systems: A Comparative Study on Ventilator-Associated Events

**DOI:** 10.3390/jcm13216600

**Published:** 2024-11-03

**Authors:** Jui-Fang Liu, Mei-Ying Kang, Hui-Ling Lin, Shih-Feng Liu

**Affiliations:** 1 Department of Respiratory Care, Chang Gung University of Science and Technology, Chiayi 61363, Taiwan; jfliu01@mail.cgust.edu.tw; 2 Chronic Diseases and Health Promotion Research Center, Chang Gung University of Science and Technology, Chiayi 61363, Taiwan; 3 Department of Respiratory Therapy, Kaohsiung Municipal Feng Shan Hospital Under the Management of Chang Gung Medical Foundation, Kaohsiung 83062, Taiwan; 4 Department of Respiratory Therapy, Chang Gung University, Taoyuan 33308, Taiwan; 5 Division of Pulmonary and Critical Care Medicine, Department of Internal Medicine, Kaohsiung Chang Gung Memorial Hospital and Chang Gung University College of Medicine, Kaohsiung 83340, Taiwan

**Keywords:** ventilator-associated event, ventilator-associated condition, infection-related ventilator condition, ventilator-associated complication, intelligent monitoring

## Abstract

**Background:** Using intelligent monitoring systems can potentially improve the identification and management of ventilator-associated events (VAEs). This single-center retrospective observational study evaluated the impact of implementing intelligent monitoring systems on the clinical outcomes of patients with VAEs in an ICU setting. **Method:** An intelligent VAE monitoring system was integrated into electronic medical records to continuously collect patient data and alert attending physicians when a ventilated patient met the criteria for a ventilator-associated condition, which was defined as an increase of at least three cm H_2_O in positive end expiratory pressure (PEEP), an increase of at least 0.20 in the fraction of inspired oxygen (FiO_2_), or the FiO_2_ being over baseline for at least two consecutive days. This study covered two phases, consisting of before using the intelligent monitoring system (2021–2022) and during its use (2023–2024). **Results:** The results showed that patients monitored with the intelligent system experienced earlier VAE detection (4.96 ± 1.86 vs. 7.77 ± 3.35 days, *p* < 0.001), fewer ventilator-associated condition (VAC) occurrences, and a shorter total duration of VACs. Additionally, the system significantly reduced ventilator days, antibiotic use, and 14-day mortality. **Conclusions:** Intelligent monitoring systems enhance VAE management, improve clinical outcomes, and provide valuable insights into the future of critical care medicine.

## 1. Introduction

Ventilator-associated events (VAEs) are common complications that can develop in patients receiving mechanical ventilation in the intensive care unit (ICU). The ratio of 10–25% was the second highest among hospital-acquired infections [1]. In a systematic review of relevant trials, ventilator-associated pneumonia (VAP) seems to have a minor impact on ICU mortality and low overall hospital-attributable mortality. However, it is linked to extended ICU stays, longer hospital admissions, and the prolonged use of mechanical ventilation [2]. These events increase patient mortality risk and significantly prolong ICU stay, thereby impacting overall healthcare quality [3,4]. Previous studies by Klompas et al. and Melsen et al. demonstrated a clear association between VAEs, elevated mortality rates, and an extended duration of mechanical ventilation [3,5].

Traditionally, VAE monitoring is conducted manually by healthcare providers. This manual approach relies on subjective judgment and manual recording by clinicians, which is time-consuming and susceptible to errors. These limitations highlight the need for more accurate and efficient monitoring tools in ICU settings. With recent advancements in medical technology, intelligent monitoring systems are increasingly being adopted to overcome these limitations. These systems automate data collection and processing and offer an objective and real-time solution for VAE monitoring.

Intelligent monitoring systems have several key advantages in ICU environments. By continuously analyzing patient data, these systems can enhance monitoring accuracy and enable the early detection of potential VAE risks, allowing for timely clinical intervention [4,6]. Research by Smyer and Shenoy et al. supports the notion that automated monitoring reduces human errors and increases the efficiency of VAE management. Additionally, the ability of these systems to provide continuous monitoring is critical, as it allows for real-time decision-making, which can significantly improve patient outcomes.

Moreover, early diagnosis and intervention facilitated by intelligent monitoring have been shown to reduce VAE-related mortality rates [6,7]. Smyer implemented an electronic algorithm for VAEs and compared it with traditional manual VAE surveillance methods. Their results showed that an electronic algorithm identified more probable VAP cases with 100% sensitivity and 100% accuracy and streamlined the workflow for infection prevention personnel [4]. Additionally, Klopmas utilized a novel surveillance system to identify VAPs, resulting in a faster diagnosis with higher sensitivity and specificity [5]. Additional intelligent systems have proven to have better sensitivity and specificity in a shorter time [8,9,10]. By automating routine tasks and reducing the reliance on subjective clinical observations, these systems not only improve the management of VAEs but also help optimize the use of healthcare resources.

Despite the theoretical advantages of intelligent monitoring systems, their clinical effectiveness and impact in real-world intensive care unit (ICU) settings remain underexplored. This study aimed to evaluate the clinical value of these systems by comparing the outcomes between patients who received VAE monitoring through intelligent systems and those who underwent traditional manual monitoring. The findings of this study provide important insights into the role of intelligent systems in improving the management of VAEs in critical care environments.

## 2. Materials and Methods

### 2.1. Implementation of the VAE Monitoring System

In January 2023, Kaohsiung Chang Gung Memorial Hospital implemented a self-developed VAE monitoring system integrated into its electronic medical records, known as the His4.0 information management system. This system continuously collects patient data and alerts attending physicians when a ventilated patient meets the criteria for a VAE. The system categorizes VAEs into three types, (1) ventilator-associated conditions (VACs), (2) infection-related ventilator-associated complications (IVACs), and (3) possible ventilator-associated pneumonia (PVAP). A VAC is defined as an increase of at least 3 cm H_2_O in the daily minimum positive end expiratory pressure (PEEP), an increase of at least 0.20 in the fraction of inspired oxygen (FiO_2_) in the daily minimum, or the FiO_2_ being over baseline for at least two consecutive days, following a minimum of two days of stable or decreasing PEEP or FiO_2_. IVAC is a subset of VAC that may be related to infection, whereas PVAP is identified by the presence of purulent secretions or positive pulmonary cultures.

### 2.2. Data Collection

This retrospective study was conducted at Kaohsiung Chang Gung Memorial Hospital, a 2700-bed tertiary care center in Southern Taiwan. The study protocol was approved by the Institutional Review Board of Chang Gung Memorial Hospital, and all methods adhered to the relevant guidelines and regulations. The requirement for informed consent was waived because this study only involved a review of the medical records and the collection of anonymized data.

Data were collected for two distinct periods, the manual VAE monitoring phase (1 August 2021 to 31 July 2022) and the intelligent automatic monitoring phase (1 August 2023 to 31 July 2024). The inclusion criteria for the study were as follows: (1) patients aged 18 years or older, (2) patients who had been on a ventilator for more than three days, and (3) patients meeting the diagnostic criteria for a VAE. The exclusion criteria included patients under 18 years of age and those without VAC events. Additionally, patients receiving extracorporeal membrane oxygenation or high-frequency ventilation were excluded, because these patients were not managed under standard ventilator settings. Consequently, alterations in the positive end expiratory pressure would not be expected despite the increased need for oxygenation.

The data collected included demographic characteristics, VAE classifications, clinical characteristics at the time of VAE occurrence, infection characteristics, and clinical outcomes.

- Demographic characteristics: data collected included the diagnosis, age, sex, weight, body mass index (BMI), acute diagnosis upon ICU admission, reason for intubation, medical history, and ICU type.
- VAE-related characteristics: the type of VAC (FiO_2_ increase by ≥0.20, PEEP increase by >3 cm H_2_O, or simultaneous increases in both FiO_2_ and PEEP), occurrences of IVAC and PVAP, Acute Physiology and Chronic Health Evaluation (APACHE) II scores at the onset and resolution of VAE, Sequential Organ Failure Assessment (SOFA) scores, the Charlson Comorbidity Index (CCI), and the number and frequency of VAC occurrences and duration were recorded.
- Infection-related characteristics: These included suspected infection sites (e.g., pulmonary, intra-abdominal, urinary, bacteremia, or unidentified infections). Pulmonary-related conditions, such as respiratory tract infections, atelectasis/sputum congestion, pulmonary embolism, pleural effusion, and aspiration pneumonia, were also documented. Extrapulmonary issues such as systemic inflammatory response syndrome, sepsis, cardiovascular complications, volume overload, heart failure, and abdominal distension were recorded.
- Clinical outcomes: this study recorded the number of days on a ventilator; extubation failures; successful ventilator liberation; antibiotic use; and 7-day, 14-day, and 90-day mortality rates.

### 2.3. Statistical Analysis

Statistical analysis was performed using SPSS version 23.0 (SSPS Inc., Chicago, IL, USA). The Kolmogorov–Smirnov test was used to assess the normality of the data distribution, and it was confirmed that all data were normally distributed. Continuous variables were presented as the mean ± standard deviation, and categorical variables were presented as counts and percentages. For the analysis of demographic data, clinical characteristics, infection characteristics, and clinical outcomes, Pearson’s chi-square test was used for categorical variables, and independent sample *t*-tests were used for continuous variables. All *p*-values were two-tailed, and *p*-values less than 0.05 were considered statistically significant.

## 3. Results

**Figure 1:**
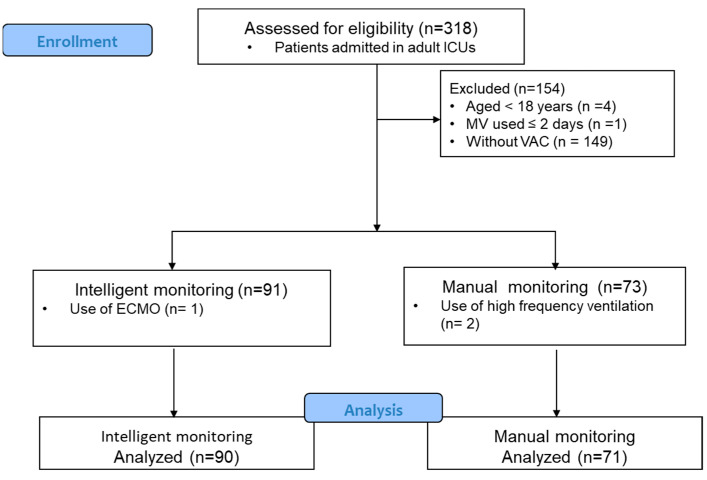
Flow chart of case enrollment in this retrospective cohort study.

During the study period, 318 patients were admitted to the adult intensive care unit (ICU). A flow chart of case enrollment is presented in Figure 1. Of these patients, 163 (approximately 52% of 318 patients) were identified as having VAEs. Prior to the implementation of intelligent automatic monitoring, 71 patients were diagnosed with VAEs via manual monitoring. After the introduction of intelligent automatic monitoring, 90 patients were identified to have VAEs.

Table 1 compares the demographic characteristics of the patients in the two groups. The characteristics of both groups were similar in terms of age, sex, admission diagnosis, medical history, reason for intubation, and type of ICU admission. Most patients (34–50%) were intubated due to impending respiratory failure. Patients admitted to medical ICUs were more likely to develop VAEs, with 76% of the cases identified in the automatic surveillance group and 48% in the manual surveillance group.

Table 2 compares the VAE classifications and clinical characteristics between the two groups. Patients in the monitored group had an earlier detection of the first VAC event after ICU admission (4.96 ± 1.86 vs. 7.77 ± 3.35 days, respectively; *p* < 0.001), experienced fewer VAC occurrences (1.37 ± 0.59 vs. 1.83 ± 0.76, respectively; *p* < 0.001), and had a shorter total number of VAC days (14.30 ± 14.19 vs. 19.14 ± 13.38 days, respectively; *p* = 0.03). Additionally, the monitored group had a higher detection rate of PVAP (46.7% vs. 29.6%, respectively; *p* = 0.03).

No significant differences were observed between the two groups in terms of the VAC detection rate, IVAC detection rate, and APACHE II score at the time of VAE occurrence or at the end of VAE; the SOFA score at the time of VAE occurrence or at the end of the VAE; the CCI at the time of VAE occurrence or at the end of the VAE; or the number of days to the first VAC event following hospital admission.

Table 3 compared the infection characteristics associated with VAEs between the two groups. No significant differences were found in the infection sites, lung-related complications, or non-lung-related complications.

Table 4 shows that compared to the manual monitoring group, the automatic monitoring group had significantly fewer days on ventilators (26.17 ± 25.24 vs. 34.14 ± 24.68, respectively; *p* = 0.046), fewer days on antibiotics (22.04 ± 22.78 vs. 32.37 ± 28.57, respectively; *p* = 0.01), and lower 14-day mortality (20.0% vs. 35.2%, respectively; *p* = 0.03). No significant differences were observed between the two groups regarding reintubation rates, successful weaning from the ventilator, 7-day mortality, or 90-day mortality.

## 4. Discussion

This intelligent system was developed to prompt physicians to intervene early when VAEs are detected. The implementation of intelligent monitoring systems for VAEs in ICUs has demonstrated substantial benefits in ventilated patients. This study revealed that these systems significantly expedite the diagnosis of VAEs, leading to an earlier detection of the first ventilator-associated complication, reduced antibiotic usage, fewer days on ventilators, and a lower 14-day mortality rate. Patients in the intelligent monitoring group experienced these advantages, with an earlier VAE detection by 3 days compared with the manual monitoring group, highlighting the system’s potential to enhance patient outcomes.

A study by Mann et al. corroborated these findings, demonstrating that intelligent monitoring systems using automated data collection can drastically reduce the time required for VAE identification [9]. Specifically, their automated VAE monitoring system evaluated 110 patients in just 1 min, whereas traditional manual methods took up to 60.7 min. Moreover, this study showed that manual monitoring missed between 18% and 54% of the cases, further emphasizing the advantage of automation in reducing human error [9].

Manual monitoring often delays the identification of VAC events by 5–13 days, which can adversely affect treatment efficiency and worsen patient outcomes. A large observational study showed 23.72 cases per 1000 ventilator days with a threefold higher hospital mortality rate [11]. Additionally, an international multicenter prospective cohort study in Europe reported 34.9 VAEs/1000 ventilator days, which occurred on the sixth day after receiving mechanical ventilation [12]. Fang et al. reported a higher mortality rate with early VAEs and longer ventilator use with late VAEs [13]. Automated systems can reduce interobserver variability by incorporating quantitative variables in electronic medical records into the VAE algorithm [14,15]. Shenoy et al. demonstrated that the sensitivity and positive predictive value of fully automated monitoring models significantly improved from 40% to 71% and 70% to 87%, respectively, highlighting the ability of automation to enhance accuracy and efficiency [5,16].

Automated VAE monitoring has also been shown to be an effective tool for improving patient care quality. Studies that integrated electronic monitoring with clinical improvement efforts during the treatment cycle demonstrated a reduced incidence of VAEs, underscoring the value of intelligent monitoring technology in enhancing clinical efficiency [17,18]. Our findings, which showed reduced ventilator days and antibiotic use in the monitored group, align with these studies.

Interestingly, our study found a higher incidence of possible ventilator-associated pneumonia in the intelligent monitoring group than in the manual monitoring group (46.7% vs. 29.6%, *p* = 0.03). This suggests that intelligent monitoring may more effectively identify potential risks in critically ill patients, thereby allowing timely intervention. Unlike traditional VAP monitoring, which relies heavily on subjective judgment, intelligent systems offer greater objectivity and specificity, thereby improving case detection rates [4]. As noted in the National Healthcare Safety Network report, the decrease in VAP prevalence in non-teaching ICUs may reflect monitoring bias rather than real improvements in nursing quality [5,16,19,20]. Our intelligent monitoring system defined PVAP as aligning with the former VAP. Consequently, subgroup analysis revealed a statistically significant difference (28.8% vs. 15.4%, *p* = 0.03). These findings indicate that the applicability of this system extends beyond the identification of VAEs.

The utility of this intelligent monitoring system extends beyond the identification of mechanical ventilation complications. It also assists in detecting severe respiratory complications and progressive underlying diseases, even when patients receive optimal care. This capacity for early identification is critical for preventing serious complications in critically ill patients [21].

However, this study had several limitations. First, the data were derived from a single medical center, which limits the generalizability of the results to other hospitals in Taiwan. Future research should consider multicenter studies and utilize the National Health Insurance database for a comprehensive nationwide analysis. Second, the two-year observation period may not be sufficient to fully evaluate the long-term effects of the intelligent monitoring system on VAEs. Additionally, operational factors, such as the proficiency and adherence of healthcare personnel in using the system, may have introduced bias into the results.

Our results indicate comparable severity scores, including APACHE and SOFA scores and the Charlson Comorbidity Index, at both the onset and conclusion of VAE occurrence between the two monitoring systems. Patients in both groups demonstrated similar baseline characteristics at the time of enrollment. VAEs were detected when the patients met the same criteria and were at similar stages in the disease process, which explains why the severity scores remained consistent across both groups. The benefits of intelligent monitoring are likely to be primarily reflected in more efficient patient management than in differences in the initial severity.

Despite these limitations, our findings suggest that deploying intelligent monitoring systems in ICUs offers significant advantages, particularly for enhancing the speed and accuracy of VAE diagnosis. Automated data collection and real-time monitoring substantially reduce the time required to detect VACs, thereby minimizing human error. Moreover, the system proved to be more efficient in the early identification and management of complications, especially in detecting PVAP, than manual methods.

The continuous monitoring capability of an intelligent system is crucial for the early detection and mitigation of potential risks, which may help prevent severe complications and reduce VAE-related mortality and healthcare costs. While these preliminary findings are promising, further validation through prospective and multicenter studies is needed to confirm the applicability and effectiveness of the system in broader clinical practice. The incidence of ventilator-associated pneumonia is notably higher in neonates and children, leading to prolonged mechanical ventilation and an increased burden on healthcare resources [22,23]. Using intelligent monitoring systems in neonatal and pediatric ICUs may offer potential benefits in reducing risk and associated burdens.

## 5. Conclusions

In conclusion, the findings of this study underscore the significance of intelligent monitoring technology in improving the accuracy and efficiency of VAE detection and management. These systems play a crucial role in enhancing the overall quality of critical care and patient safety. However, while the results are promising, future research should focus on prospectively validating these findings across multiple centers to assess the external applicability of intelligent monitoring systems. Such studies would provide a more comprehensive evaluation of the impact of these technologies on clinical outcomes and their potential to improve ICU practices on a broader scale.

## Figures and Tables

**Table 1:** Comparisons of demographic characteristics.

Parameters	Intelligent Monitoring (*n* = 90)	Manual Monitoring (*n* = 71)	*p*-Value
Age (years)	66.6 ± 13.96	68.46 ± 14.04	0.42 ^b^
BMI (kg/m^2^)	25.07 ± 6.18	28.58 ± 24.31	0.18 ^b^
Gender; man, *n* (%)	25.07 ± 6.18	28.58 ± 24.31	0.18 ^b^
Diagnosis at admission			
Acute kidney injury	11(12.2)	13 (18.3)	0.28 ^a^
Congestive heart failure	8 (8.9)	7 (9.9)	0.83 ^a^
Cerebrovascular disease	8 (8.9)	4 (5.6)	0.43 ^a^
Chronic liver disease	14 (15.6)	12 (16.9)	0.83 ^a^
COPD	11 (12.2)	10 (14.1)	0.52 ^a^
Collagen disease	4 (4.4)	2 (2.8)	0.69 ^a^
DM	12 (13.3)	13 (18.3)	0.39 ^a^
Ischemic heart disease	8 (8.9)	6 (8.5)	0.92 ^a^
Malignancy	10 (11.1)	14 (19.7)	0.18 ^a^
Medical history			0.47 ^a^
Heart disease, *n* (%)	5 (5.6)	3 (4.2)	
Liver disease, *n* (%)	9 (10.0)	5 (7.0)	
Renal disease, *n* (%)	24 (26.7)	20 (28.2)	
Hematologic diseases, *n* (%)	24 (26.7)	18 (25.4)	
Respiratory disease, *n* (%)	15 (16.7)	15 (21.1)	
Diabetes mellitus, *n* (%)	10 (11.1)	6 (8.5)	
Cerebrovascular disease, *n* (%)	3 (3.3)	4 (5.6)	
Reason for intubation			0.66 ^a^
Inability to keep airway open, *n* (%)	0	1 (1.4)	
Failure to protect airway from aspiration, *n* (%)	8 (8.9)	4 (5.6)	
Ventilation failure, *n* (%)	4 (4.4)	2 (2.8)	
Insufficiency in oxygenation, *n* (%)	14 (15.6)	12 (16.9)	
Possible conditions that may lead to respiratory failure, *n* (%)	31 (34.4)	36 (50.7)	
Other, *n* (%)	33 (36.7)	16 (22.5)	
Type of ICU			0.55 ^a^
GSICU, *n* (%)	12 (13.3)	16 (22.5)	
CVSICU, *n* (%)	5 (5.5)	3 (4.2)	
NSICU, *n* (%)	6 (6.6)	4 (5.6)	
NICU, *n* (%)	15 (16.6)	4 (5.6)	
MICU, *n* (%)	38 (42.2)	30 (42.3)	
RICU, *n* (%)	24 (26.6)	14 (19.8)	

^a^: Chi-square test; ^b:^ independent sample *t*-test. Values are expressed as numbers (%) or mean ± standard deviation. Abbreviations: BMI—body mass index; COPD—chronic obstructive pulmonary disease; DM—diabetes mellitus; GSICU—general surgical intensive care unit; CVSICU—cardiovascular surgical intensive care unit; NSICU—neurosurgical intensive care unit; NICU—neurology intensive care unit; MICU—medical intensive care unit; RICU—respiratory intensive care unit.

**Table 2:** Comparisons of VAE classification and clinical characteristics.

Parameters	Intelligent Monitoring (*n* = 90)	Manual Monitoring (*n* = 71)	*p*-Value
Incidence of VAC			
Increased FiO_2_ > 0.2, *n* (%)	17 (18.9)	17 (23.9)	0.44 ^a^
Increased PEEP 9 cm H_2_O, *n* (%)	81 (90.0)	52 (81.7)	0.17 ^a^
Increased both FiO_2_ 0.2 and PEEP 3 cm H_2_O, *n* (%)	21 (23.3)	10 (14.1)	0.16 ^a^
Number of IVAC occurrences, *n* (%)	51 (56.7)	33 (46.5)	0.21 ^a^
Number of PVAP occurrences, *n* (%)	42 (46.7)	21 (29.6)	0.03 * ^a^
APACE score at the time of VAE occurrence	29.01 ± 10.7	28.72 ± 9.82	0.86 ^b^
APACE score at the end of the VAE	22.02 ± 6.35	22.01 ± 5.99	0.99 ^b^
SOFA score at the time of VAE occurrence	10.89 ± 7.4	10.48 ± 5.38	0.69 ^b^
SOFA score at the end of the VAE	6.76 ± 2.9	7.55 ± 3.48	0.13 ^b^
CCI score at the time of VAE occurrence	6.26 ± 3.14	6.59 ± 3.34	0.51 ^b^
CCI score at the end of the VAE	5.53 ± 2.65	5.70 ± 2.81	0.69 ^b^
Days of VAC occurred from admission	25.97 ± 23.61	26.73 ± 18.74	0.82 ^b^
Days of first VAC detection following ICU admission	4.96 ± 1.86	7.77 ± 3.35	<0.001 ** ^b^
Number of VAC occurrence	1.37 ± 0.59	1.83 ± 0.76	<0.001 ** ^b^
Number of VAC days	14.3 ± 14.19	19.14 ± 13.38	0.03 * ^b^

^a^: Chi-square test; ^b^: independent sample *t*-test; * *p* < 0.05. ** *p* < 0.01. Abbreviations: VAE—ventilator-associated event; VAC—ventilator-associated condition; IVAC—infection-related ventilator-associated complication; VAP—ventilator-associated pneumonia; CCI—Charlson Comorbidity Index; SOFA—Sequential Organ Failure Assessment.

**Table 3:** Comparisons of infection characteristics associated with VAEs.

Parameters	Intelligent Monitoring (*n* = 90)	Manual Monitoring (*n* = 71)	*p*-Value
Site of infection			
Pulmonary, *n* (%)	66 (73.3)	43 (60.6)	0.44 ^a^
Intra-abdominal, *n* (%)	19 (21.1)	10 (14.1)	0.31 ^a^
Urinary tract, *n* (%)	18 (20.1)	23 (32.4)	0.07 ^a^
Bacteremia, *n* (%)	16 (17.8)	16 (22.5)	0.55 ^a^
Unidentified infection, *n* (%)	5 (5.6)	2 (2.8)	0.47 ^b^
Intrapulmonary-related			0.39 ^a^
None, *n* (%)	24 (26.7)	28 (39.4)	
Respiratory tract infection, *n* (%)	24 (26.7)	14 (19.7)	
Atelectasis/sputum plug, *n* (%)	15 (16.7)	13 (18.3)	
Pneumothorax, *n* (%)	7 (7.8)	3 (4.2)	
Pulmonary embolus, *n* (%)	2 (2.2)	1 (1.4)	
Pleural effusion, *n* (%)	15 (16.7)	7 (12.7)	
Aspiration, *n* (%)	3 (3.3)	5 (7.0)	
Extrapulmonary-related			0.39 ^a^
None, *n* (%)	31 (34.4)	32 (45.1)	
New onset of SIRS/sepsis, *n* (%)	26 (28.9)	20 (28.2)	
Cardiac/circulatory, *n* (%)	8 (8.9)	4 (5.6)	
Volume overload, *n* (%)	7 (7.8)	9 (7.1)	
Heart failure, *n* (%)	11 (12.2)	2 (2.8)	
Abdominal distension, *n* (%)	4 (4.4)	1 (1.4)	
No reason for VAC identified, *n* (%)	3 (3.3)	3 (4.2)	

^a^: Chi-square test; ^b^: independent sample *t*-test;

**Table 4:** Comparisons of clinical outcomes.

Parameters	Intelligent Monitoring (*n* = 90)	Manual Monitoring (*n* = 71)	*p*-Value
Ventilator days	26.17 (25.24)	34.14 (24.68)	0.04 * ^b^
Extubation failure	0.67 (0.75)	0.79 (1.05)	0.062 ^b^
Reintubation, *n* (%)	30 (33.3)	31 (43.7)	0.19 ^a^
Successful weaning from the ventilator, *n* (%)	71 (78.9)	50 (70.4)	0.27 ^a^
Days of antibiotic use	22.04 (22.78)	32.37 (28.57)	0.01 * ^b^
7-day mortality, *n* (%)	8 (8.9)	12 (16.9)	0.15 ^a^
14-day mortality, *n* (%)	18 (20.0)	25 (35.2)	0.03 * ^a^
90-day mortality, *n* (%)	20 (44.4)	25 (55.6)	0.79 ^a^

^a^: Chi-square test; ^b^: independent sample *t*-test; * *p* < 0.05.

## Data Availability

The data supporting the findings of this study are available from the corresponding author (S.-F.L.) upon reasonable request.
